# Unveiling the genetic architecture of barley embryo: QTL mapping, candidate genes identification and its relationship with kernel size and early vigour

**DOI:** 10.1007/s00122-025-04817-y

**Published:** 2025-01-23

**Authors:** Xiaoxia Chen, Zhouyang Su, Yunpu Zheng, Cong Li, Jun Ma, Jian Ma, Fusun Shi, Haiyan Hu, Chunji Liu, Zhi Zheng

**Affiliations:** 1 CSIRO Agriculture and Food, 2 Clunies Ross Street, Acton, ACT 2601 Australia; 2 Chendu Institute of Biology, Chinese Academy of Sciences, Chengdu, 610213 China; 3 School of Water Conservancy and Hydropower, Hebei University of Engineering, Handan, 056006 China; 4 State Key Laboratory of Crop Gene Exploration and Utilization in Southwest China, Triticeae Research Institute, Sichuan Agricultural University, Chengdu, 611130 China; 5 College of Agronomy and Biotechnology, China Agricultural University, Beijing, 100193 China; 6 College of Agriculture, Henan Institute of Science and Technology, Xinxiang, 453003 Henan China

## Abstract

In this first QTL mapping study of embryo size in barley, novel and stable QTL were identified and candidate genes underlying a significant locus independent of kernel size were identified based on orthologous analysis and comparison of the whole-genome assemblies for both parental genotypes of the mapping population.

Embryo, also known as germ, in cereal grains plays a crucial role in plant development. The embryo accounts for only a small portion of grain weight but it is rich in nutrients. Larger embryo translates to a more nutritious grain and larger store of energy reserves, which can benefit seed germination and seedling establishment. However, reports on quantitative trait loci (QTL) affecting embryo size in barley is rare. To understand the genetic basis of embryo size in barley, a population consisting of 201 F9 recombination inbred lines (RILs) was assessed in four environments. Three regions affecting various characteristics of embryo size including embryo length (EL), embryo width (EW) and embryo area (EA) were consistently identified. They located on chromosomes 2H, 4H and 7H, respectively. Among them, the QTL on 7H was not significantly affected by kernel size. Phenotypic variances explained by this QTL for EL, EW and EA were 11.8%, 9.3% and 12.7%, respectively. Taken advantage of the available genomic assemblies of the two parental genotypes, candidate genes for this locus on 7H were identified. In addition, significant correlations between embryo size and early vigour and kernel traits were detected. To our knowledge, the present study is for the first time reporting QTL conferring embryo size by directly measuring the characteristics as quantitative trait in barley, which would broaden our understanding of the genetic basis of barley embryo size and offer valuable targets for future breeding programmes.

**Supplementary Information:**

The online version contains supplementary material available at 10.1007/s00122-025-04817-y.

## Introduction

Barley, ranked as the fourth most significant cereal crop in terms of both production and economic impact, serves as a crucial component in stockfeed and forms the foundation for malting and brewing (Walker et al. 2013). Cereal grains consist of four main components: the hull or husk, the bran layer, the endosperm, and the embryo or germ. The embryo constitutes a relatively small portion (2–3%) of the total kernel weight but it is packed with essential nutrients that are vital for plant growth and development including protein, vitamins, oil and non-starch carbohydrates (Raj et al. 2023). Increasing relative embryo size is known to enhance grain quality and showed nutritional benefits for human food and animal feed (Lee et al. 2016; Jung et al. 2017). As the demand for high-quality grains surges, cereal varieties with larger embryos present an exciting avenue for enhancing the nutritional value of our food sources (Orman-Ligeza et al. 2020). While embryo size is not the sole determinant of yield, it can significantly impact grain weight and overall crop productivity.

Embryo size has been a key indicator for agronomic improvement on early vigour (Zhao et al. 2019). Results from previous studies showed that larger embryos in cereals are associated with faster seed germination, greater crop establishment, increased leaf width, enhanced seedling shoot growth and improved root development (Forbis et al. 2002; Richards and Lukacs 2002; Rebetzke et al. 2008; Maydup et al. 2012; Finch-Savage and Bassel 2016; Hendriks et al. 2022). Rebetzke et al. (2022) suggested that these traits increase resource capture and weed competitiveness, further improving crop performance and grain yield under water-limited conditions (Li et al. 2019; Rebetzke et al. 2022). While embryo size is associated with kernel size (López‐Castañeda et al. 1996; Richards and Lukacs 2002; Li et al. 2022), it is suggested that embryo size independent of kernel size may be an important determinant of final number and size of seminal roots (Rebetzke et al. 2022). In barley, embryo size is an important determinant of early viability (López‐Castañeda et al. 1996; Moore and Rebetzke 2015). Therefore, understanding of genetic architecture for barley embryo size and its genetic association to kernel size and early vigour would accelerate the improvement of barley quality and seedling establishment.

Linkage mapping is one of the main approaches for identifying loci for a targeted trait. Compared to extensive investigations in other cereal crops such as rice, maize and wheat (Moore and Rebetzke 2015; Lee et al. 2019; Yuan et al. 2019; Li et al. 2022; Rebetzke et al. 2022; Chen et al. 2023; Katral et al. 2023; Wang et al. 2023), the genetic studies on barley embryo size is rare. The only genetic study on this trait in barley was based on a mutagenized germplasm described by Orman-Ligeza et al. (2020). In that study, the embryo size was assessed as a qualitative trait using an F2 population. A high-lysine locus (*LYS3*) encoding a prolamin-box-binding transcription factor was identified as controlling embryo size on chromosome arm 5HL. However, there have been few attempts to elucidate the genetic basis of embryo size based on natural variation. Despite of the small size of the embryo, its enclosure within the hull poses significant challenges for direct measurement, hindering genetic studies on embryo in hulled barley.

In this study, we for the first time attempted to identify novel QTL conferring embryo size including embryo length (EL), embryo width (EW) and embryo area (EA) in barley. To achieve this, seeds from a population consisting of 201 F9 recombinant inbred lines (RILs) were harvested and assessed across four field sites during the 2022 and 2023 seasons. In addition, we investigated its correlations with kernel size and early vigour traits. Moreover, we employed comparative analysis of genes with known effects in other crop species like rice between the two parental genotypes. This approach has proven to be powerful for identifying candidate genes within targeted regions (Gao et al. 2024; Zheng et al. 2022; Zhou et al. 2021). Leveraging the high-quality genome assemblies available for the two parental genotypes of this population, we successfully predicted candidate genes underlying a locus independent of kernel size. The findings of this research are detailed in this publication.

## Materials and methods

### Plant materials

A RIL population consisting of 201 F9 lines was assessed in this study. This RIL population was generated from a cross between Morex, a six-row malting barley, and AWCS276, a two-row wild barley (Zhou et al. 2021). Single seed descendent method and fast generation procedure were used to advance this population (Zheng et al. 2013).

### Phenotypic assessment

Data on embryo size were collected from four field trials. The mapping population, along with the two parents, were assessed in each of the field trials. The field trials were carried out at CSIRO Forest Hill Research Station (27°33′S, 152°16′E) and Boorowa Research Station (34°47′S, 148°41′E) in 2022 and 2023. These trials were designated as FH22, FH23, BO22 and BO23, respectively. Each trial contained two replicates, with each replicate consisting of ten seedlings planted at a spacing of 20 cm within a single row. Row was spaced 25 cm apart. Field management was according to local agricultural practices.

At Zadoks 92, five well-pollinated spikes from each line were harvested from the main tillers of each plant and put into a 37 °C oven for four days. Eight well-developed kernels from the middle sections of central spikelets from each of the five spikes were selected and then carefully removed the hull without damaging the embryos. Then, the hump of barley kernels was adhered to double-sided tape in Petri dishes to facilitate the measurement. Embryo characteristics including EL and EW were measured using a stereomicroscope (OLYMPUS-SZX7; Olympus, Tokyo, Japan) as described by Wang et al. (2023). The embryo area (EA) is calculated as EA = EL*EW*0.72 as described by Moore and Rebetzke (2015). Kernel characteristics of the same kernels, including kernel length (KL), kernel width (KW) and kernel area (KA), were measured using an SC6000R digital image analyser (Next Instruments, Condell Park, Australia). Maximum root length of seedlings (MRL) was determined by measuring the length of the longest root from crown to root tip at 14 days by growing the seedlings in water-moisture paper rolls. Thousand kernel weight (TKW) and early vigour traits including coleoptile length (CL), third leaf thickness of seedling (S3LT), third leaf length of seedlings (S3LL), third leaf width of seedling (S3LW), third leaf area (S3LA), and ratios of third leaf length and width of seedlings (S3LWR) were retrieved from previous studies (Zheng et al. 2022; Gao et al. 2024).

### Statistical analysis

The average values of each line in a single environment and the best linear unbiased prediction (BLUP) value estimated from average values of different environments were used for QTL identification and further analysis. BLUP of target traits and the broad-sense heritability (*H*^*2*^) were calculated using the PROC MIXED and VARCOMP procedures of SAS V8.0 (SAS Institute, Cary, NC, USA; https://www.sas.com). SPSS18.0 software (SPSS, Chicago, IL, USA) was used to perform normal distribution test, Student’s t test (*P* < *0.05*) and correlation analysis of phenotype values in different trials.

### QTL analysis

A high-density genetic map of the mapping population based on genotyping by sequencing (GBS) data was constructed according to the previous study (Zhou et al. 2021). A total of 1,140 polymorphic markers were used in this study, resulting in the linkage map spanning approximately 1022.4 cM with an average marker distance of 0.7 cM. MapQTL 6.0 (Van Ooijen and Kyazma 2009) was employed to identify putative QTL conferring embryo size. A test of 1000 permutations were performed to identify the LOD threshold corresponding to a genome-wide false discovery rate of 5% (*P* < 0.05) for each trial. Interval mapping was then used to identify QTL and MapChart was used to draw linkage maps with QTL positions (Voorrips 2002). Confidence interval is defined as the region on either side of the QTL peak where the LOD score drops by 1.5 compared to the peak LOD score (Visscher et al. 1996). The flanking markers were identified as the nearest markers bordering the support interval.

### Conditional QTL analysis

To understand the complex relationships between embryo size and other agronomic traits, conditional QTL analysis was employed using QGAStation 2.0 software (http://ibi.zju.edu.cn/software/) (Zhu 1995) to identify QTL while considering the influence of other traits. For example, A|B indicates that A is conditional on B. BLUP datasets were used to generate the conditional phenotypic values. The interpretation of conditional QTL results can be categorized into four scenarios: (1) QTL for A detected only in traditional analysis, suggesting that B influences A through this locus (2) QTL for A detected in both non-conditional and conditional analyses with similar effect values. This indicates that the locus affects trait A independently; (3) QTL for A detected in both non-conditional and conditional analyses, with significant changes in effect values, which implies that the locus impacts both A and B simultaneously; and (4) QTL for A detected only in conditional analysis, indicating that the effect of this locus on A is masked by B.

### Identification of candidate genes underlying QTL for embryo size

Candidate genes were identified in the targeted QTL independent of kernel size as described by Zheng et al. (2022). Briefly, sequences of two markers flanking the confidence interval were used to blast against genome assemblies of barley pseudomolecules Morex (Mascher et al. 2021) to define the physical interval. Coding sequence and protein sequences of predicted genes in the identified QTL regions were retrieved from ftp://ftp.ensemblgenomes.org/pub/release-44/plants/gff3/hordeum_vulgare for Morex and NCGR wild barley database http://db.ncgr.ac.cn/wild_barley/ for AWCS276 (Liu et al. 2020). Gene sequences known to related to embryo and kernel size in rice were used to identify potentially homologous genes with the Morex genome assembly. Candidate genes detected in Morex were then blasted against the genome assembly of AWCS276 for differences. Reciprocal best hits analysis using DIAMOND v2.0 (Buchfink et al. 2021) was performed to understand the potential functional roles of the identified protein sequences.

## Results

### Phenotypic data analysis in the mapping population

**Fig. 1:**
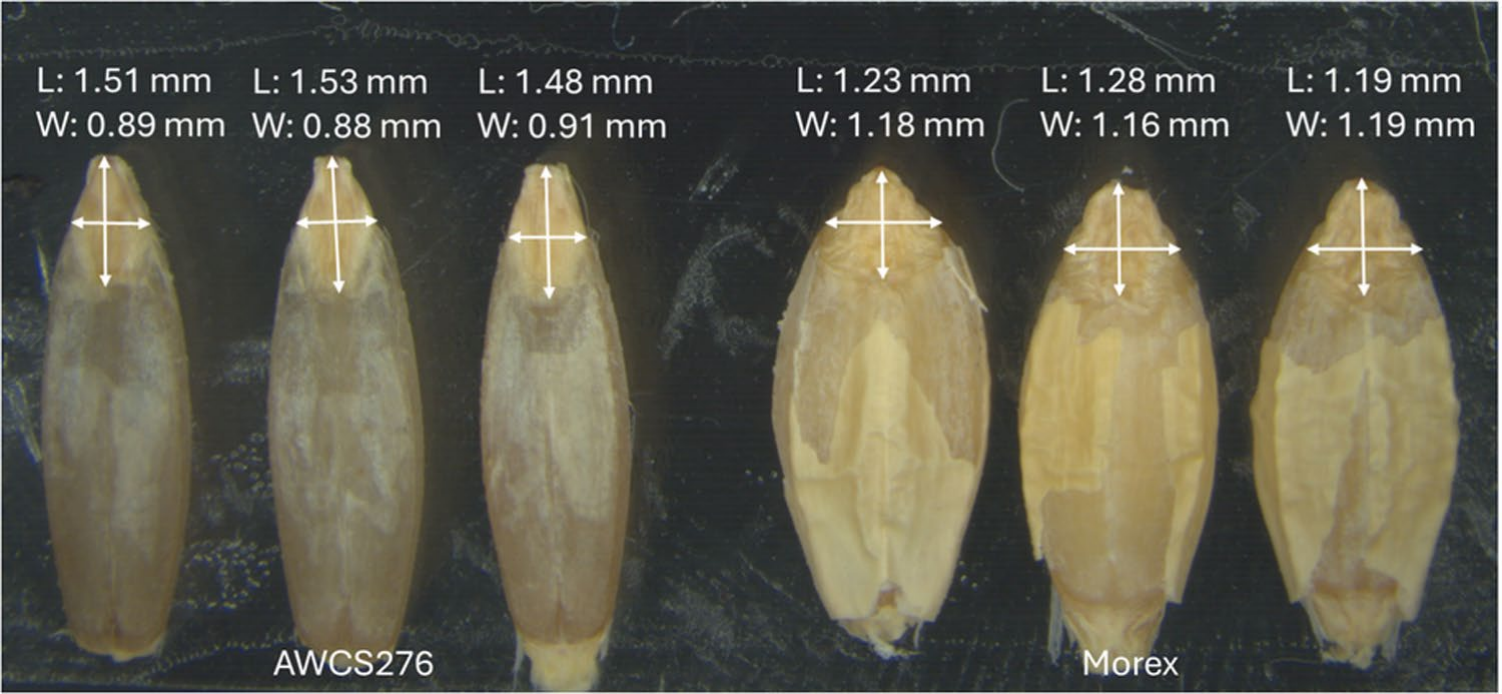
Morphologies of the two parental genotypes, AWCS276 and Morex, showing the differences in embryo length (L) and width (W)

**Fig. 2:**
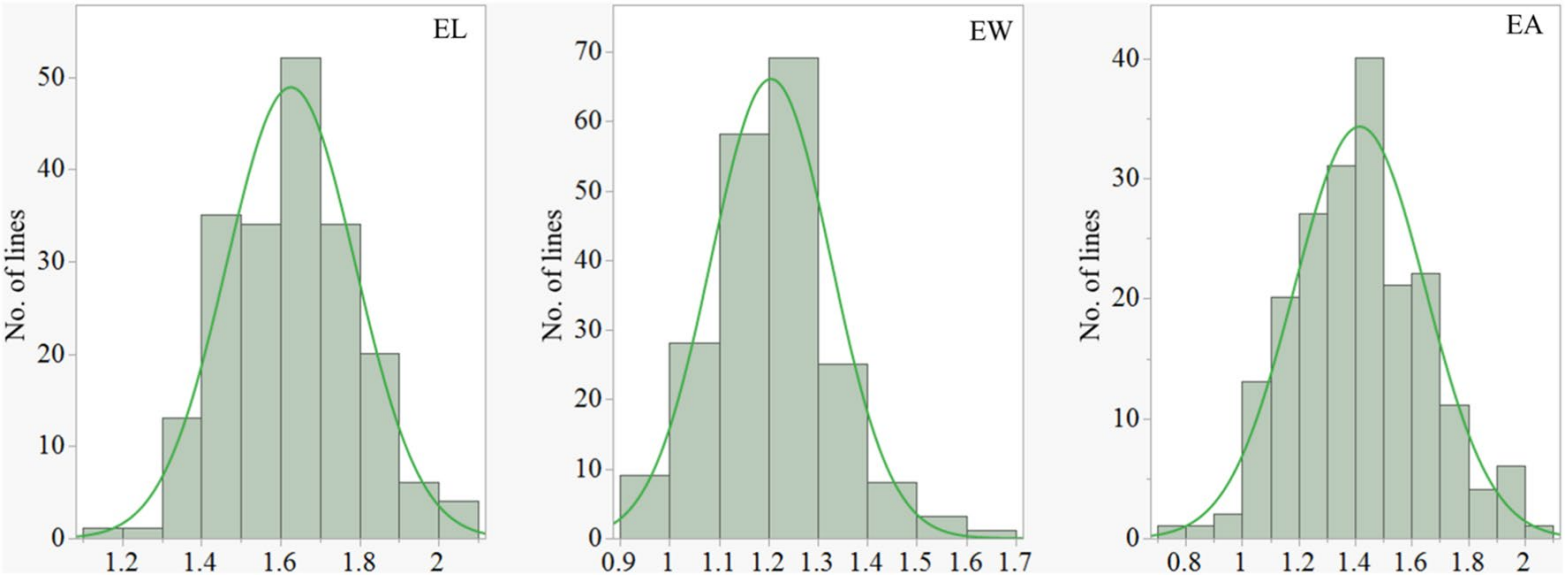
Frequency distributions for embryo length (EL), embryo width (EW), and embryo area obtained from the population of Morex/AWCS276 using BLUP datasets

Values of the three embryo size traits (EL, EW and EA) showed highly significant and positive correlation across four different trials and the BLUP dataset (*P* < *0.01*), with correlation coefficients varying from 0.76 to 0.98 (Table S1). In all four trials conducted, AWCS276 had significantly greater EL but smaller EW and EA compared to Morex (Fig. 1; Table 1). The observed variation within the mapping population for EL ranged from 1.18 to 2.05 mm and the standard deviation (SD) of 0.16 mm in the BLUP dataset, while the EW varied from 0.91 to 1.65 mm with the SD of 0.12 mm in the BLUP dataset (Table 1). The *H*^*2*^ estimates for EL, EW and EA were 0.82, 0.90 and 0.89, respectively. These high heritability values suggest that genetic factors played major roles in determining these embryo characteristics (Table 1). Moreover, the frequency distribution of all these three traits displayed continuous variation with transgressive segregation (Fig. 2).

**Table 1:** Phenotypic variation and heritability of embryo size for the parents and population assessed in different environments

#Trait	Trial	Parents		Population					
		Morex	AWCS276	Min	Max	Mean	SD	CV(%)	H^2^
EL(mm)	FH22	1.23	1.51	1.17	2.04	1.62	0.17	10.20	
	BO22	1.28	1.53	1.18	2.06	1.63	0.17	10.34	
	FH23	1.19	1.58	1.17	2.04	1.62	0.17	10.23	
	BO23	1.22	1.52	1.17	2.08	1.63	0.17	10.51	
	BLUP	1.24	1.55	1.18	2.05	1.63	0.16	10.03	0.82
EW(mm)	FH22	1.18	0.92	0.91	1.63	1.21	0.12	10.26	
	BO22	1.16	0.88	0.91	1.64	1.20	0.13	10.41	
	FH23	1.19	0.91	0.92	1.63	1.21	0.12	10.28	
	BO23	1.21	0.89	0.89	1.65	1.21	0.13	10.47	
	BLUP	1.20	0.90	0.91	1.64	1.21	0.12	10.07	0.90
EA(mm^2^)	FH22	1.18	0.93	0.80	2.01	1.42	0.24	16.72	
	BO22	1.21	0.96	0.79	2.01	1.41	0.24	16.86	
	FH23	1.15	1.00	0.79	2.02	1.42	0.24	16.74	
	BO23	1.08	0.88	0.79	2.01	1.42	0.24	17.04	
	BLUP	1.13	0.96	0.80	2.01	1.42	0.23	16.41	0.89

^*#*^*EL* embryo length, *EW* embryo width, *EA* embryo area, *FH22*, *BO22*, *FH23* and *BO23* four independent trials conducted for embryo size, *BLUP* best linear unbiased prediction, *SD* standard deviation, *CV* coefficient of variation, *H*^*2*^ the broad-sense heritability

### Correlations between embryo size and kernel size and early vigour traits

Significant and positive relationships were detected among all three embryo characteristics (*P* < *0.001*). Potential correlations between embryo size and 11 other traits of agronomic importance were also analysed using the BLUP datasets (Table 2). These agronomic traits included early vigour traits (CL, MRL, S3LT, S3LL, S3LW, S3LA, and S3LWR) and kernel traits (KL, KW, KA and TKW), which were collected from independent experiments using the same population. All three embryo characteristics showed positive and significant correlations with all four kernel traits, except for EL which showed no correlation with KW and TKW (Table 2). In addition, both EW and EA were significantly and positively correlated with S3LL, S3LA, MRL and TKW, while EW was also significantly associated with S3LW, EL and EA were positively correlated with S3LWR. Furthermore, none of the three embryo traits showed significant correlations with S3LT and CL (Table 2).

**Table 2:** Coefficients of pairwise Pearson correlations between embryo size and other traits of agronomic importance

Group	Traits	EL	EW	EA
Embryo traits	EL	1.00		
	EW	0.35***	1.00	
	EA	0.82***	0.83***	1.00
Kernel traits	KL	0.41***	0.18*	0.39***
	KW	0.02^ ns^	0.54***	0.34***
	KA	0.29***	0.47***	0.46***
	TKW	0.03^ ns^	0.42***	0.27***
Early vigour traits	S3LT	–0.04^ ns^	-0.03^ ns^	-0.04^ ns^
	S3LL	0.12^ ns^	0.14*	0.16*
	S3LW	–0.07^ ns^	0.12*	0.03^ ns^
	S3LA	0.06^ ns^	0.16*	0.13*
	S3LRW	0.20**	0.06^ ns^	0.17**
	MRL	0.12^ ns^	0.21**	0.20**
	CL	0.10^ ns^	0.01^ ns^	0.06^ ns^

^#^Correlations were calculated using BLUP datasets. *EL* embryo length, *EW* embryo width, *EA* embryo area, *KL* kernel length, *KW* kernel width, *KA* kernel area, *TKW* thousand kernel weight S*3LT* 3rd leaf thickness from seedling, *S3LL* 3rd leaf length from seedling, *S3LW* 3rd leaf width from seedling, *S3LA* 3rd leaf area from seedling, *S3LWR* 3rd leaf length and width ratio from seedling, *MRL* maximum root length, *CL* coleoptile length,

Note: ‘ns’, ‘*’, ‘**’, and ‘***’ refer to significance of correlations (*ns* not significant, *P* < *0.05, P* < *0.01, P* < *0.001*)

### QTL identification for embryo size

Assessment of the whole mapping population detected three putative QTL regions associated with all three embryo characteristics on chromosomes 2H, 4H and 7H. The allele for large embryo size of the 7H locus was derived from the parent Morex, and the alleles for large embryo size of the 2H and 4H loci were derived from AWCS276. These QTL were consistently detected in all four trials as well as the BLUP dataset (Table 3). The most significant QTL for EL was located on chromosome 7H (designated as *Qes.caf-7H*) explaining up to 11.8% of the phenotypic variance with a maximum LOD value of 5.2, while the largest effect QTL targeting EW was identified on 2H (designated as *Qes.caf-2H*) accounting for 15.6% of the phenotypic variance with a LOD value of 7.1. Regarding EA, the largest effect QTL was detected on the short arm of chromosome 4H (designated as *Qes.caf-4H*), which explained the phenotypic variance ranging from 13.7 to 14.8% with the LOD values varying from 6.2 to 6.7 (Fig. S1, Table 3).

**Table 3:** QTL for embryo size identified in the population of Morex/AWCS276#

Traits	Trials	QTL	Chr	Linkage map interval (cM)	Physical map interval (Mbp)	LeftMarker	RightMarker	LOD	PVE (%)	Add
EL	FH22	*Qes.caf-2H*	2H	52.1–59.3	529.1–542.6	GBS_MST812	GBS_MST1258	4.0	9.2	–0.050
		*Qes.caf-4H*	4H	15.8–20.9	13.2–23.0	GBS_MST2470	GBS_MST2490	3.4	7.8	–0.046
		*Qes.caf-7H*	7H	89.8–96.3	597.9–615.1	GBS_MST4580	GBS_MST4562	5.2	11.8	0.057
	BO22	*Qes.caf-2H*	2H	52.9–59.5	529.1–542.6	GBS_MST828	GBS_MST1262	3.3	7.7	–0.047
		*Qes.caf-4H*	4H	15.8–20.9	13.2–23.0	GBS_MST2470	GBS_MST2490	3.0	6.8	–0.044
		*Qes.caf-7H*	7H	89.8–96.6	597.9–615.1	GBS_MST4580	GBS_MST4560	4.6	10.4	0.055
	FH23	*Qes.caf-2H*	2H	53.2–59.3	529.1–542.6	GBS_MST833	GBS_MST1258	3.7	8.7	–0.049
		*Qes.caf-4H*	4H	15.8–24.9	13.2–24.3	GBS_MST2470	GBS_MST2491	3.4	7.7	–0.046
		*Qes.caf-7H*	7H	89.5–96.3	597.9–615.1	GBS_MST4580	GBS_MST4562	5.1	11.4	0.057
	BO23	*Qes.caf-2H*	2H	52.9–59.5	529.1–542.6	GBS_MST821	GBS_MST1262	3.0	6.9	–0.045
		*Qes.caf-4H*	4H	12.9–23.3	11.2–24.3	GBS_MST2469	GBS_MST2490	2.8	6.6	–0.044
		*Qes.caf-7H*	7H	91.5–98.8	597.9–615.1	GBS_MST4573	GBS_MST4554	4.1	9.3	0.053
	BLUP	*Qes.caf-2H*	2H	52.9–59.3	529.1–542.6	GBS_MST828	GBS_MST1258	3.5	8.2	–0.047
		*Qes.caf-4H*	4H	12.9–24.9	11.5–24.3	GBS_MST2469	GBS_MST2491	3.1	7.2	–0.044
		*Qes.caf-7H*	7H	89.5–96.3	597.9–615.1	GBS_MST4580	GBS_MST4562	4.8	10.7	0.055
EW	FH22	*Qes.caf-2H*	2H	67.4–72.0	568.8–586.1	GBS_MST1320	GBS_MST1355	7.1	15.6	–0.049
		*Qes.caf-4H*	4H	15.8–19.8	13.2–22.7	GBS_MST2470	GBS_MST2477	6.4	14.2	–0.047
		*Qes.caf-7H*	7H	72.7–91.2	571.7–603.8	GBS_MST4649	GBS_MST4574	4.0	9.1	0.037
	BO22	*Qes.caf-2H*	2H	67.4–71.7	568.8–586.1	GBS_MST1320	GBS_MST1352	6.9	15.3	–0.049
		*Qes.caf-4H*	4H	15.8–19.8	13.2–22.7	GBS_MST2470	GBS_MST2477	6.5	14.5	–0.048
		*Qes.caf-7H*	7H	76.9–85.9	581.1–593.4	GBS_MST4621	GBS_MST4586	4.1	9.3	0.038
	FH23	*Qes.caf-2H*	2H	67.4–72.0	568.8–586.1	GBS_MST1320	GBS_MST1355	7.0	15.5	–0.049
		*Qes.caf-4H*	4H	15.8–19.8	13.2–22.7	GBS_MST2470	GBS_MST2477	6.4	14.2	–0.047
		*Qes.caf-7H*	7H	74.6–85.9	578.2–593.4	GBS_MST4633	GBS_MST4586	4.0	9.1	0.037
	BO23	*Qes.caf-2H*	2H	67.4–71.7	568.8–584.1	GBS_MST1320	GBS_MST1352	7.2	15.8	–0.050
		*Qes.caf-4H*	4H	15.8–19.8	13.2–22.7	GBS_MST2470	GBS_MST2477	6.3	14.0	–0.047
		*Qes.caf-7H*	7H	76.9–85.9	581.1–593.4	GBS_MST4621	GBS_MST4586	4.1	9.2	0.039
	BLUPUP	*Qes.caf-2H*	2H	67.4–72.0	568.8–586.1	GBS_MST1320	GBS_MST1355	7.1	15.5	–0.049
		*Qes.caf-4H*	4H	15.8–19.8	13.2–22.7	GBS_MST2470	GBS_MST2477	6.4	14.2	–0.047
		*Qes.caf-7H*	7H	76.9–85.9	581.1–593.4	GBS_MST4621	GBS_MST4586	4.1	9.2	0.038
EA	FH22	*Qes.caf-2H*	2H	54.9–60.3	529.1–542.6	GBS_MST1160	GBS_MST1264	5.4	12.2	–0.083
		*Qes.caf-4H*	4H	15.8–19.8	13.2–22.7	GBS_MST2470	GBS_MST2477	6.6	14.7	–0.091
		*Qes.caf-7H*	7H	91.5–94.5	602.9–613.1	GBS_MST4573	GBS_MST4562	5.6	12.7	0.086
	BO22	*Qes.caf-2H*	2H	54.2–61.4	529.1–542.6	GBS_MST1049	GBS_MST1274	4.7	10.7	–0.078
		*Qes.caf-4H*	4H	15.8–19.8	13.2–22.7	GBS_MST2470	GBS_MST2477	6.5	14.3	–0.090
		*Qes.caf-7H*	7H	91.5–96.6	602.9–613.1	GBS_MST4573	GBS_MST4560	5.0	11.4	0.082
	FH23	*Qes.caf-2H*	2H	54.9–60.3	529.1–542.6	GBS_MST1160	GBS_MST1264	5.2	11.6	–0.080
		*Qes.caf-4H*	4H	15.8–19.8	13.2–22.7	GBS_MST2470	GBS_MST2477	6.7	14.8	–0.091
		*Qes.caf-7H*	7H	91.5–96.3	602.9–613.1	GBS_MST4573	GBS_MST4562	5.4	12.1	0.084
	BO23	*Qes.caf-2H*	2H	54.9–62.0	529.1–542.6	GBS_MST1160	GBS_MST1276	4.5	10.3	–0.077
		*Qes.caf-4H*	4H	15.8–19.8	13.2–22.7	GBS_MST2470	GBS_MST2477	6.2	13.7	–0.089
		*Qes.caf-7H*	7H	91.5–96.3	602.9–613.1	GBS_MST4573	GBS_MST4562	4.5	10.3	0.079
	BLUP	*Qes.caf-2H*	2H	54.9–61.4	529.1–542.6	GBS_MST1160	GBS_MST1274	5.0	11.2	–0.079
		*Qes.caf-4H*	4H	15.8–19.8	13.2–22.7	GBS_MST2470	GBS_MST2477	6.5	14.4	–0.090
		*Qes.caf-7H*	7H	91.5–96.3	602.9–613.1	GBS_MST4573	GBS_MST4562	5.2	11.6	0.082

^*#*^*EL* embryo length, *EW* embryo width, *EA* embryo area, *Chr* chromosome, FH22, BO22, FH23 and BO23 four independent trials conducted for embryo size, *BLUP* best linear unbiased prediction, *cM* centimorgan, *PVE* phenotype variance explained, *Add* Additive effect (positive values: alleles for long coleoptile from Morex, negative values: alleles for long coleoptile from AWCS276)

### Conditional QTL detection for embryo size

**Fig. 3:**
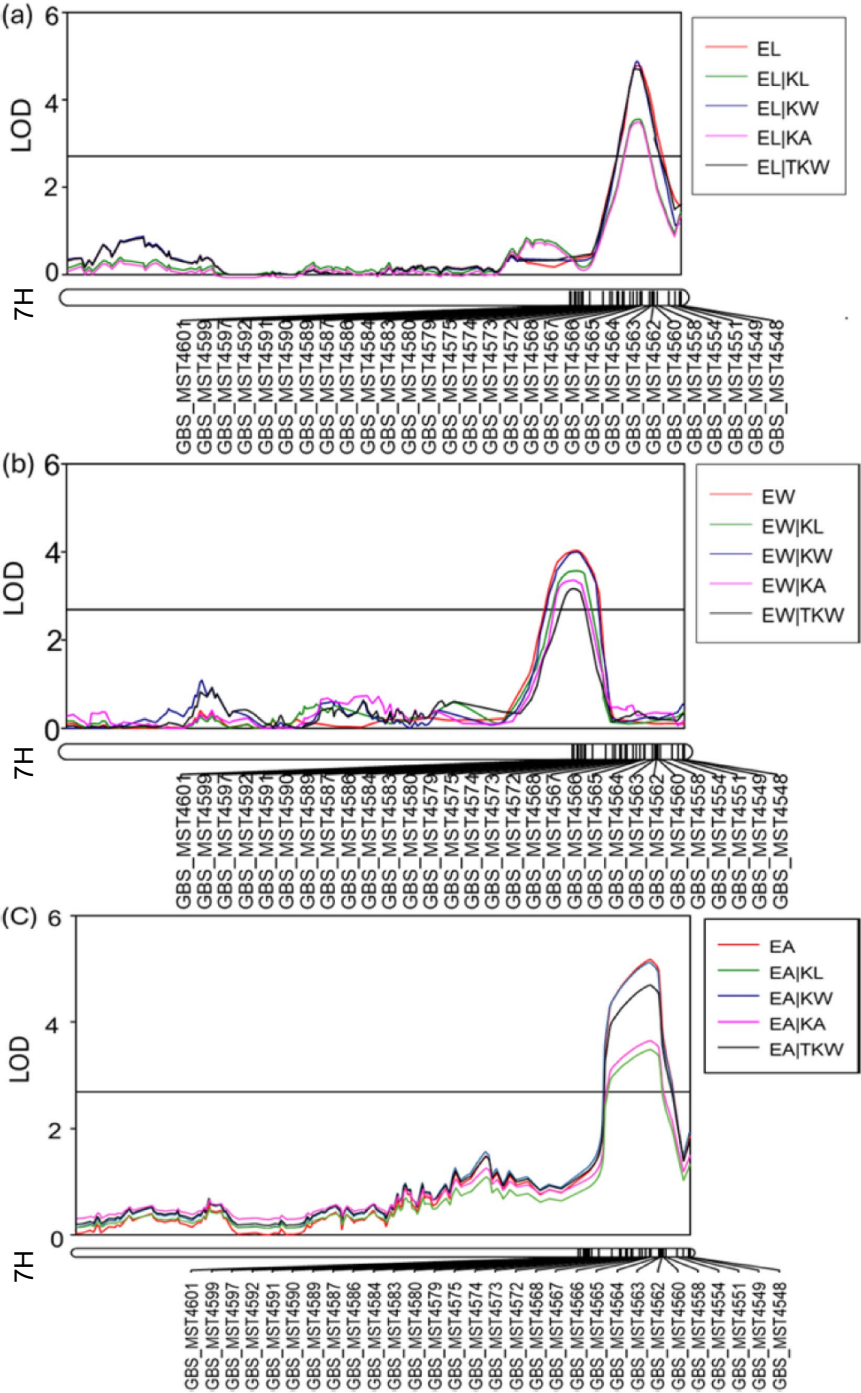
Conditional QTL analysis of *Qes.caf-7H* with kernel-related traits and 1000-kernel weight, showing the changes of LOD value when **a** embryo length (EL), **b** embryo width (EW) and **c** embryo area (EA) was conditional on kernel length (KL), kernel width (KW), kernel area (KA) and 1000-kernel weight (TKW), respectively

Conditional QTL analysis was conducted using the BLUP datasets and identified five QTL regions associated with embryo size, including three regions previously detected in the traditional QTL analysis and two newly discovered regions. For all three embryo characteristics, *Qes.caf-7H* remained significant with slight changes in LOD values and phenotypic variation explained (PVE) when conditioned on all kernel traits (Fig. 3). This result suggested that this QTL was potentially independent of kernel traits. *Qes.caf-2H* and *Qes.caf-4H* showed no significant effects on EL when conditioned on KL and KA but remained significant when conditioned on KW and TKW, suggesting that these two QTL were probably dependent on KL and KA and independent of KW and TKW. Regarding EW and EA, while the LOD values of *Qes.caf-4H* did not change significantly (when they were conditional on all kernel traits), those of *Qes.caf-2H* decreased dramatically (LOD < 2.7) and only remain significant on EW when conditional on KL (Table S2). Two QTL conferring EW, one conferring EL and one QTL conferring EA were newly identified on chromosomes 1H and 6H when removing the effects of KW, KA and TKW. These results indicated that the effects of the newly detected QTL were masked by these kernel traits (Fig. S1, Table S2).

### Candidate genes underlying the major locus on chromosome 7H

**Fig. 4:**
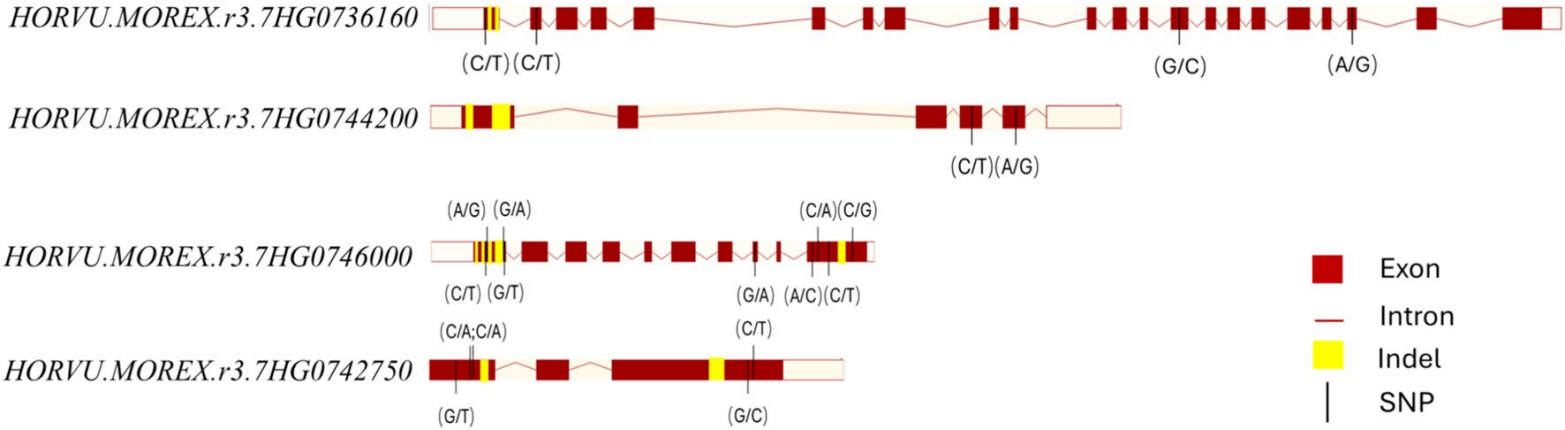
Gene structures and genome sequence alignment of candidate genes for *Qes.caf-7H* between Morex and AWCS276

Sequences for 488 genes associated with embryo and kernel size from rice were obtained and blasted against the assemblies of Morex and AWCS276 (Table S3). This orthologous analysis identified four candidate genes in the 7H region, including *HORVU.MOREX.r3.7HG0736160*, *HORVU.MOREX.r3.7HG0742750*, *HORVU.MOREX.r3.7HG0744200*, and *HORVU.MOREX.r3.7HG0746000*. Genomic comparation of the two parental genotypes detected insertion-deletion (Indel) in all the four candidate genes (Fig. 4). *HORVU.MOREX.r3.7HG0736160* contained two deletions (39 bp and 48 bp) and one SNP variation in the first exon and one SNP substitution in each of the exon 2, 14 and 20, respectively (Fig. 4). Two deletions (one 27 bp and the other one 51 bp) were identified in exon 1 of *HORVU.MOREX.r3.7HG0744200* between the two parental genotypes, resulting in a 9-amino acid deletion and an 18-amino acid deletion. When comparing the two parents, four Indels (3 bp, 6 bp, 3 bp and 39 bp, respectively) and four SNP variations in the first exon as well as one SNP in the eighth exon and one Indel and three SNPs in the tenth exon were observed from *HORVU.MOREX.r3.7HG0746000*. The fourth gene *HORVU.MOREX.r3.7HG0742750* in AWCS276 contained a 3 bp insertion and three SNP variations in the first exon as well as a 6 bp deletion and two SNP variations in the third exon when compared to Morex. Importantly, all these Indels between the parents resulted in alterations to the protein domain structure and integrity.

## Discussion

Embryo size is a critical trait influencing early seedling vigour, germination rate, overall crop establishment and quality in cereal crops. Despite its importance, the genetic control of embryo size remains poorly understood in barley. To date, only one gene associated embryo size has been reported as a qualitative trait on chromosome 5H derived from a mutagenized barley germplasm (Orman-Ligeza et al. 2020). Thus, all the QTL identified in this study are novel and have not been previously reported. Unlike that study, we for the first time identified three novel QTL regions associated with embryo size from natural variation using direct measurements in barley. Among these QTL, the one located on 7HL chromosome arm remained significant after removing the effects of kernel size. Further comparative genomic analysis identified four candidate genes in the 7H interval. These genes all contain large Indels leading to amino acid changes, making them primary targets for future cloning efforts to identify the gene(s) underlying this locus. Our study provides valuable insights into the genetic architecture of barley embryo size and offers promising targets for future research and breeding efforts.

Unlike the oblique orientation of wheat embryo, barley embryo lies relatively flat within the seed. However, most barley is ‘covered barley’, which has a tough, inedible outer hull surrounding the barley kernel. This hull is beneficial for yield and is in high demand for malting due to its role in the malting process (Hebelstrup et al. 2017; Raj et al. 2023), nevertheless the presence of this hull poses a significant challenge for understanding the complex genetics underlying embryo size. To overcome this challenge, we carefully removed the hull from eight well-developed kernels, primarily located in the centre of the spike to enable accurate measurement of embryo size. This method, previously used to identify major and stable QTL conferring embryo size in wheat (Wang et al. 2023), was slightly modified for barley. By adhering the hump of barley kernels to double-sided tape in Petri dishes, we were able to effectively measure embryo size using stereomicroscope and identified consistent QTL conferring this trait.

Given that the embryo is part of seed, it is understandable that significantly positive correlations were detected between them. This suggests that selecting for larger seeds can lead to larger embryos (Wang et al. 2023). However, conditional QTL analysis revealed that *Qes.caf-7H* remained significant with only slight changes in LOD values and PVE when removing the effects of kernel size, suggesting its potential independence from kernel size. Therefore, *Qes.caf-7H* could be a valuable target for further improving embryo size in the breeding programs. Moreover, the present study also detected significantly positive relationships between embryo size and early vigour traits. For example, EW was significantly correlated with S3LL, S3LW, S3LA and MRL (Table 2). Previous studies have reported that larger embryos are associated with increased leaf width and seedling root growth (Rebetzke et al. 2008; Maydup et al. 2012; Richards and Lukacs 2002; Hendriks et al. 2022). As reported by Rebetzke et al. (2022), embryo size independent of kernel size may be a crucial determinant of the final number and size of seminal roots in wheat. By incorporating the novel QTL identified in this study, breeding programs can potentially improve resource capture, weed competitiveness, and ultimately improved crop performance and grain yield under water-limited conditions (Li et al. 2019; Rebetzke et al. 2022).

Orthologous and comparative analysis identified four candidate genes within the *Qes.caf-7H* region: *HORVU.MOREX.r3.7HG0736160*, *HORVU.MOREX.r3.7HG0744200, HORVU.MOREX.r3.7HG0746000, HORVU.MOREX.r3.7HG0742750*. These genes are orthologous to *TWINKLE/RECA3*, *HOS59*, *AP2-4 and GW6a*/*OsglHAT1* in rice, respectively. It has been reported that *TWINKLE/RECA3* interacting with *OsmtSSB1* impacted kernel size and weight (Li et al. 2021), which may in turn impact embryo size due to their correlations. *HOS59*, a rice plant knotted1-like homeobox (KNOX) Class II subfamily gene, is known to regulate grain size and embryonic/postembryonic development in various plants (Tsuda and Hake 2015; Sheng et al. 2022). Previous studies have also reported *AP2* gene family mediates kernel size by affecting embryo, endosperm and seed coat development in Arabidopsis (Jofuku et al. 2005; Ohto et al. 2005, 2009). In addition, *GW6a*/*OsglHAT1* in rice is associated with kernel length and weight, encoding a novel GNAT-like protein with intrinsic histone acetyltransferase activity (Song et al. 2015). It was shown that *GW6a*/*OsglHAT1* interacts with *HDR3* (a ubiquitin-containing receptor) regulating kernel size by positively regulating cell number and grain filling rate (Gao et al. 2021). Further investigation is warranted to elucidate the precise functions of these candidate genes and their interactions in regulating embryo development.

## Supplementary Information

Below is the link to the electronic supplementary material.

Supplementary file1 (XLSX 30 kb)

Supplementary file2 (DOCX 203 kb)

## Data Availability

The datasets generated during and/or analysed during the current study are available from the corresponding author upon reasonable request.
