# Supplementary material for: Unveiling the genetic architecture of barley embryo: QTL mapping, candidate genes identification and its relationship with kernel size and early vigour
Source: Theor Appl Genet. 2025 Jan 23;138(1):32. doi: 10.1007/s00122-025-04817-y (PMC11754356; doi:10.1007/s00122-025-04817-y)
Supplement: Supplementary file 2 — Supplementary file2 (DOCX 203 kb) [file 122_2025_4817_MOESM2_ESM.docx]

**Table S1.** Correlation coefficients of embryo size in the population of Morex/AWCS276 among the four trials and BLUP dataset#

| Trait | EL_FH22 | EL_BO22 | EL_FH23 | EL_BO23 | EL_BLUP |
| --- | --- | --- | --- | --- | --- |
| EL_FH22 | 1.00 |  |  |  |  |
| EL_BO22 | 0.98** | 1.00 |  |  |  |
| EL_FH23 | 0.96** | 0.92** | 1.00 |  |  |
| EL_BO23 | 0.88** | 0.95** | 0.94** | 1.00 |  |
| EL_BLUP | 0.85** | 0.91** | 0.81** | 0.82** | 1.00 |
|  | EW_FH22 | KW_BO22 | KW_FH23 | KW_BO23 | EA_BLUP |
| EW_FH22 | 1.00 |  |  |  |  |
| EW_BO22 | 0.82** | 1.00 |  |  |  |
| EW_FH23 | 0.76** | 0.71** | 1.00 |  |  |
| EW_BO23 | 0.81** | 0.98** | 0.87** | 1.00 |  |
| EW_BLUP | 0.91** | 0.93** | 0.87** | 0.94** | 1.00 |
|  | EA_FH22 | EA_BO22 | KA_FH23 | KA_BO23 | EA_BLUP |
| EA_FH22 | 1.00 |  |  |  |  |
| EA_BO22 | 0.93** | 1.00 |  |  |  |
| EA_FH23 | 0.97** | 0.76** | 1.00 |  |  |
| EA_BO23 | 0.92** | 0.98** | 0.81** | 1.00 |  |
| EA_BLUP | 0.95** | 0.97** | 0.84** | 0.94** | 1.00 |

#*EL* embryo, *EW* embryo width, *EA* embryo area, *FH22, BO22, FH23 and BO23* four independent trials conducted for embryo size, *BLUP* best linear unbiased prediction, ** significant at *P* *< 0.01*

**Table S2.** Conditional QTL analysis-based determination of relationships between embryo and other agronomic traits

| Traits | Chr. | Linkage map interval (cM) | Physical map interval (Mbp) | LeftMarker | RightMarker | LOD | PVE (%) |
| --- | --- | --- | --- | --- | --- | --- | --- |
| EL\|KL | 7H | 89.8-96.3 | 597.9-615.1 | GBS_MST4580 | GBS_MST4562 | 3.5 | 7.9 |
| EL\|KW | 2H | 52.9-59.5 | 529.1-542.6 | GBS_MST828 | GBS_MST1262 | 3.3 | 7.7 |
|  | 4H | 15.8-20.9 | 13.2-23.0 | GBS_MST2470 | GBS_MST2490 | 3.1 | 7.1 |
|  | 7H | 89.8-96.6 | 597.9-615.1 | GBS_MST4580 | GBS_MST4560 | 4.7 | 10.7 |
| EL\|KA | **6H** | **33.6-38.2** | **274.5-327.2** | **GBS_MST4430** | **GBS_MST4093** | **3.0** | **6.9** |
|  | 7H | 89.5-96.3 | 597.9-615.1 | GBS_MST4580 | GBS_MST4562 | 3.4 | 7.8 |
| EL\|TKW | 2H | 52.9-59.5 | 529.1-542.6 | GBS_MST821 | GBS_MST1262 | 3.2 | 7.4 |
|  | 4H | 12.9-23.3 | 13.2-23.0 | GBS_MST2469 | GBS_MST2490 | 3.1 | 7.2 |
|  | 7H | 91.5-98.8 | 597.9-615.1 | GBS_MST4573 | GBS_MST4554 | 4.7 | 10.5 |
| EW\|KL | 2H | 67.4-72.0 | 568.8-586.1 | GBS_MST1320 | GBS_MST1355 | 5.7 | 12.7 |
|  | 4H | 12.9-24.9 | 11.2-25.7 | GBS_MST2469 | GBS_MST2491 | 5.4 | 12.0 |
|  | 7H | 76.9-85.9 | 581.1-593.4 | GBS_MST4621 | GBS_MST4586 | 3.5 | 8.1 |
| EW\|KW | 4H | 15.8-19.8 | 13.2-22.7 | GBS_MST2470 | GBS_MST2477 | 6.3 | 14.8 |
|  | **6H** | **33.6-38.2** | **274.5-327.2** | **GBS_MST4430** | **GBS_MST4093** | **3.4** | **7.9** |
|  | 7H | 76.9-85.9 | 581.1-593.4 | GBS_MST4621 | GBS_MST4586 | 4.1 | 9.2 |
| EW\|KA | 4H | 15.8-19.8 | 13.2-23.0 | GBS_MST2470 | GBS_MST2477 | 4.2 | 10.1 |
|  | 7H | 76.9-85.9 | 581.1-593.4 | GBS_MST4621 | GBS_MST4586 | 3.3 | 7.5 |
| EW\|TKW | **1H** | **67.4-72.0** | **493.3-512.4** | **GBS_MST625** | **GBS_MST648** | **3.0** | **6.9** |
|  | 4H | 15.8-19.8 | 13.2-22.7 | GBS_MST2470 | GBS_MST2477 | 7.2 | 15.9 |
|  | 7H | 76.9-85.9 | 581.1-593.4 | GBS_MST4633 | GBS_MST4586 | 3.1 | 7.1 |
| EA\|KL | 4H | 15.8-19.8 | 13.2-22.7 | GBS_MST2470 | GBS_MST2477 | 4.8 | 10.8 |
|  | 7H | 76.9-85.9 | 602.9-613.1 | GBS_MST4621 | GBS_MST4586 | 3.6 | 8.2 |
| EA\|KW | 4H | 15.8-19.8 | 13.2-22.7 | GBS_MST2470 | GBS_MST2477 | 5.7 | 13.1 |
|  | **6H** | **33.6-38.2** | **274.5-327.2** | **GBS_MST4430** | **GBS_MST4093** | **3.4** | **7.7** |
|  | 7H | 91.5-94.5 | 602.9-613.1 | GBS_MST4573 | GBS_MST4562 | 5.1 | 11.5 |
| EA\|KA | 4H | 15.8-19.8 | 13.2-22.7 | GBS_MST2470 | GBS_MST2477 | 4.3 | 10.6 |
|  | **6H** | **33.6-38.2** | **274.5-327.2** | **GBS_MST4430** | **GBS_MST4093** | **3.6** | **8.3** |
|  | 7H | 91.5-94.5 | 602.9-613.1 | GBS_MST4573 | GBS_MST4562 | 3.5 | 7.9 |
| EA\|TKW | 4H | 15.8-19.8 | 13.2-22.7 | GBS_MST2470 | GBS_MST2477 | 6.7 | 14.7 |
|  | **6H** | **33.6-38.2** | **274.5-327.2** | **GBS_MST4430** | **GBS_MST4093** | **2.8** | **6.6** |
|  | 7H | 91.5-94.5 | 602.9-613.1 | GBS_MST4573 | GBS_MST4562 | 4.6 | 10.4 |

The QTL in bold indicates that the QTL newly detected in conditional QTL analysis, and the remaining QTL without bold indicates that can be detected both in conditional and traditional QTL analysis.


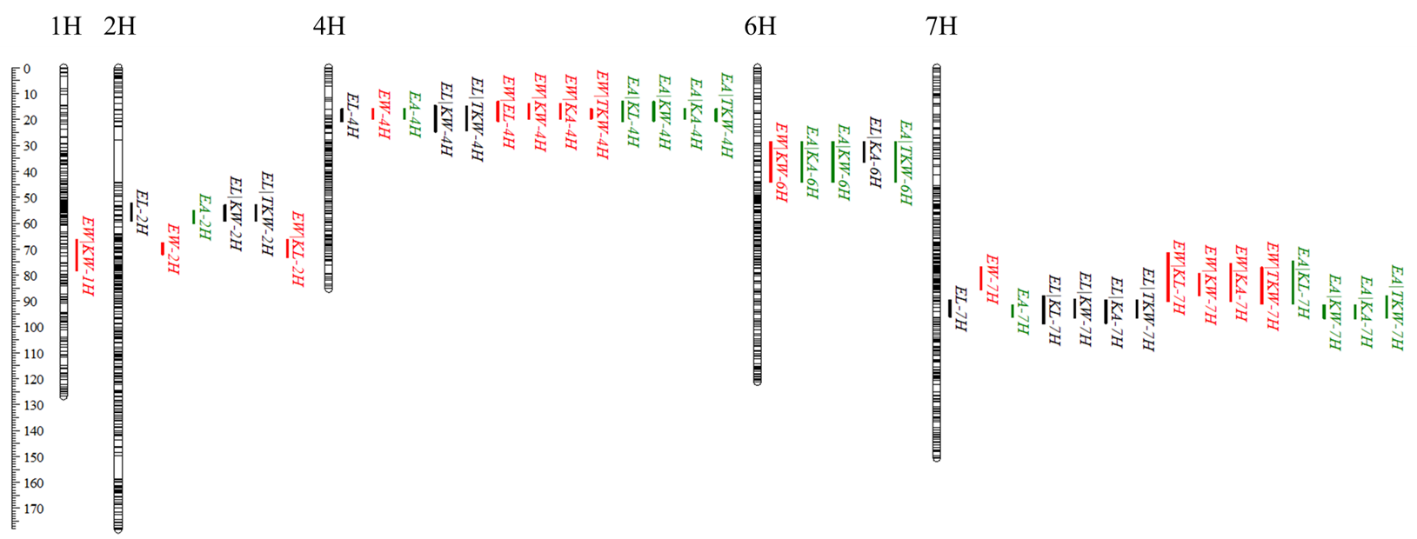


**Figure S1.** QTL for embryo size identified from both traditional and conditional QTL analysis in the population of Morex/AWCS276 using BLUP datasets. *EL* embryo length, *EW* embryo width, *EA* embryo area, *KL* kernel length, *KW* kernel width, *KA* kernel area, *TKW* thousand kernel weight.
